# FILIAL PIETY AND RELATIONSHIP QUALITY AMONG OLDEST-OLD PARENTS AND OLDER-ADULT CHILDREN IN KOREA: A DYADIC APPROACH

**DOI:** 10.1093/geroni/igz038.3424

**Published:** 2019-11-08

**Authors:** Kyuho Lee, Joohong Min, Gyounghae Han

**Affiliations:** 1 Daegu University, Gyeongsan, Korea, Republic of; 2 Jeju National University, Jeju, Korea, Republic of; 3 Seoul National University, Seoul, Korea, Republic of

## Abstract

Objectives: Research has consistently reported the importance of adult children’s filial piety in maintaining relationship quality with their older-adult parents, especially in Asian countries due in large part to the influence of Confucianism. However, less attention has been paid to its effects in their later relationship—oldest-old parents and older-adult children, and very few studies employed a dyadic approach. Methods: In the current study, we examined the effects of actor- and partner effects of the attitudes toward filial piety on intergenerational relationship quality. Data from 105 dyads of very old parents, 81 to 97 years old (M = 87.9, SD = 2.8) and their older-adult children, 65 to 72 years old (M = 65.9, SD = 1.2) were utilized for the analyses. Results: Results showed that children’s attitudes toward filial piety were positively related to the relationship quality reported by both old children (i.e., actor-effects, β = .64, p < .001) and oldest-old parents (i.e., partner-effects, β = .63, p = .001). The effect of actor’s and partner’s attitudes toward filial piety on relationship quality was not significant. Discussion: Findings suggest that children’s attitudes toward filial piety, as compared to parents, may play a more important role in the quality of relationships between oldest-old parents and their older-adult children in South Korea.

